# Seroprevalence of Toscana Virus and Sandfly Fever Sicilian Virus in European Bat Colonies Measured Using a Neutralization Test

**DOI:** 10.3390/v13010088

**Published:** 2021-01-11

**Authors:** Nazli Ayhan, Marc López-Roig, Abir Monastiri, Remi N. Charrel, Jordi Serra-Cobo

**Affiliations:** 1 Unité des Virus Emergents, Aix Marseille University, IRD 190, INSERM U1207, 13005 Marseille, France; nazliayhann@gmail.com (N.A.); remi.charrel@univ-amu.fr (R.N.C.); 2 UR7310, Laboratoire de Virologie, Université de Corse-Inserm, 20250 Corte, France; 3 Department of Evolutionary Biology, Ecology and Environmental Sciences, Faculty of Biology, University of Barcelona, 08028 Barcelona, Spain; mlroig@gmail.com (M.L.-R.); abirmonastiri@gmail.com (A.M.); 4 Biodiversity Research Institute, University of Barcelona, 08028 Barcelona, Spain

**Keywords:** phlebovirus, transmission, reservoir, meningitis, arbovirus

## Abstract

Toscana phlebovirus (TOSV) and Sicilian phlebovirus (SFSV) are endemic in the Mediterranean area where they are transmitted to humans by infected sandflies. Vertebrates of several species have been postulated to act as reservoirs of these viruses, but convincing evidence is still awaited. Among them, bats have been suggested, however documented evidence is lacking. Here we tested a total of 329 bats belonging to eight species collected from twelve localities in southern Spain for the presence of neutralizing antibodies specific to TOSV and SFSV. Positive sera were detected in Schreiber’s long-fingered bat (*Miniopterus schreibersii*)*,* mouse-eared Myotis (*Myotis myotis*), European free-tailed bat (*Tadarida teniotis*), and common serotine (*Eptesicus serotinus*) with the latter showing the highest prevalence rates for SFSV (22.6%) and TOSV (10%). There was no difference between females and males. Results suggest that bats are not likely to play a major role in the natural cycle of these two sandfly-borne phleboviruses. However, large breeding colonies of bats can be used as sentinels for surveillance of the presence of such viruses in a given locality. In addition, capture–recapture studies should be initiated in order to understand better the dynamics of TOSV and SFSV in bat populations.

## 1. Introduction

Toscana virus (TOSV) and sandfly fever Sicilian virus (SFSV) are members of the Toscana phlebovirus and Sicilian phlebovirus species (genus Phlebovirus, family Phenuiviridae, order Bunyavirales). TOSV and SFSV are endemic in the Mediterranean area where Toscana (TOSV) and sandfly fever Sicilian (SFSV) viruses can infect humans through the bite of infected female sandflies. After a 3–5 days incubation period, the SFSV infection consists of an onset of abrupt and severe fever, headaches, malaise, photophobia, myalgia, and retro-orbital pain. The duration of the fever is 2–3 days. Leucopenia can be observed during the onset of the disease. Infections consist of an acute febrile illness (3 to 5 days) with fever, myalgia, and headaches. TOSV has a marked tropism for central and peripheral neurological systems and is an important cause of acute aseptic meningitis and meningoencephalitis during the warm season. Virus clinical infection starts as a mild febrile illness, following an incubation period of 3–7 days, without the involvement of the central nervous system (CNS). Neuroinvasive infections usually begin with a headache, fever, nausea, vomiting, and myalgia. Physical examination may show neck rigidity, Kernig sign, and in some cases unconsciousness, tremors, paresis, and nystagmus. [1,2]. Sandflies that are able to transmit these viruses belong to the genus phlebotomus, which has a Palearctic distribution. Sandflies have crepuscular or nocturnal activity in places with high relative humidity and an absence of wind [3]. TOSV and SFSV antibodies were found in horses, cats, dogs, sheep, pigs, cattle, and goats [1,4]. Vertebrates of several species have been postulated to act as reservoirs of these viruses, but convincing evidence is still awaited. The role of dogs in the maintenance of these viruses has been recently excluded based on experimental studies [5]. Bats harbor a significantly higher proportion of zoonotic viruses than all other mammalian orders: more than 200 viruses of 28 families have been isolated or detected in bats [6,7]. They have been recognized as important reservoirs of zoonotic viruses worldwide. Very little is known about the role of bats in the ecology of phleboviruses transmitted by sandflies. For these reasons, we performed a prospective neutralization-based seroprevalence study of TOSV and SFSV in bat colonies located in Spain.

## 2. Methods

### 2.1. Sample Collection

**Figure 1:**
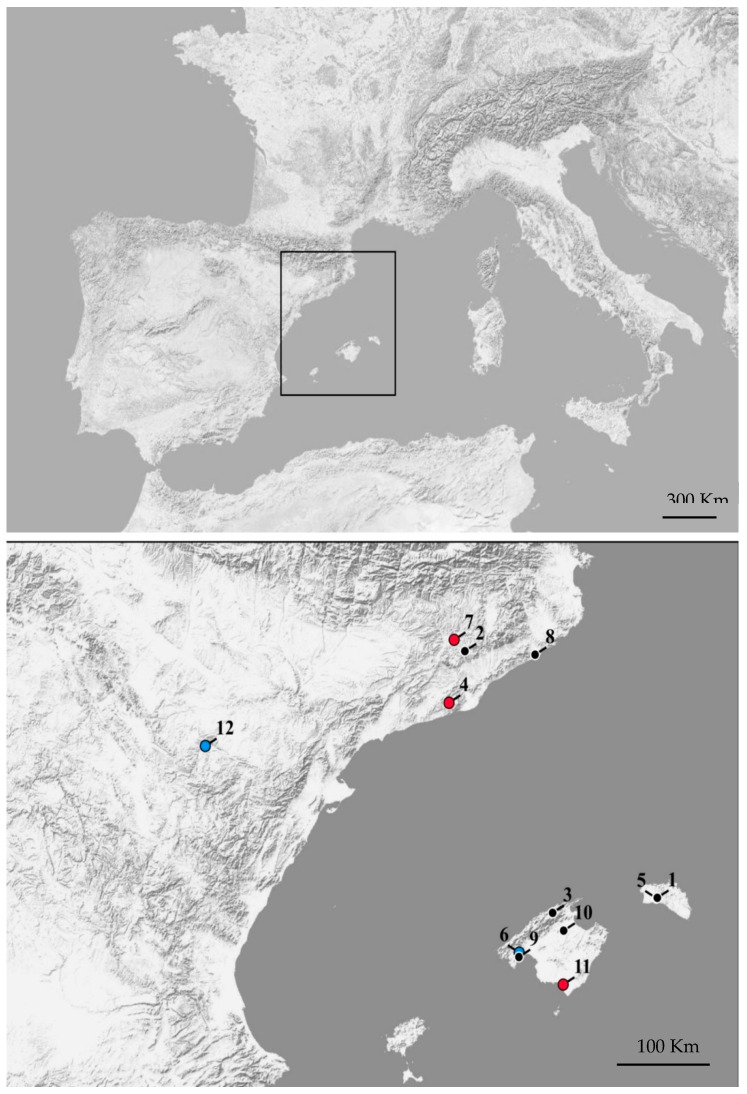
Map of north-eastern Spain showing the sampled localities. Blue circles indicate localities where TOSV-seropositive bats were found. Red circles indicate localities where TOSV- and SFSV-seropositive bats were found. Black circles correspond to localities where all samples were negative.

From 2006 to 2018, blood samples of bats were collected from 12 localities in three Spanish autonomous regions: Aragon, the Balearic Islands, and Catalonia (Figure 1). Bat colonies were sampled throughout the year, except during the hibernation (from mid-December to the end of February) and birthing (from mid-June to mid-July) periods. Insectivorous bats were captured inside the roosts with long-handled butterfly nets during the day or with mist nets at sunset, when they emerge to forage. The morphological identification of bats was performed in compliance with the identification keys for the bats of Europe, and the bats were sexed. Thick leather gloves were worn when the bats were handled and transferred into individual cotton pouches for transportation and processing. Blood samples (30–250 μL, depending on the individual size, and based on a volume <1% of body mass) were obtained by a small puncture made next to the proximal epiphysis of the radius in compliance with the Food and Agriculture Organization guidelines. Samples were stored at 4 °C for a few hours before undergoing 12,100 g centrifugation for 20 min. Serum and clot pellets were separated and stored at −20 °C until further processed [8].

### 2.2. Detection of Neutralizing Antibodies Against TOSV and SFSV

Each serum was analyzed for SFSV-NT-Abs first, and then for TOSV-NT-Abs if the sample volume was sufficient. The virus microneutralization (MN) assay of this study was adapted from the protocol described previously [9]. Sera were tested in parallel for TOSV (strain MRS2010–4319501) and SFSV (strain Sabin). Sera were heat-inactivated at 56 °C for 30 min. The MN assay was performed in 96-well microtiter plates using Vero cells. Briefly, two-fold serial dilutions of 50 µL serum aliquots were mixed with an equal volume of 100 TCID50 (tissue culture infective dose producing pathological change in 50% of the cell culture inoculated) of viruses into 96-well plates, providing two-fold final dilutions between 1:20 and 1:160. Controls consisted of each serum (1:10) with Vero cells but without the virus. After five days (for TOSV) and six days (for SFSV), the microplates were read and the presence (neutralization titer at 20, 40, 80, and 160) or absence (no neutralization) of the cytopathic effect was noted. The cutoff value for positivity was set at titer equal to or higher than 40, as previously described using the same technique [10,11]. However, an alternative criterion of positivity including also titers at 20 has been considered [9,12]. Results using both criteria (titer ≥ 40 and titer ≥ 20) are presented in Table 1, Table 2 and Table 3 but only results obtained using the most stringent criteria (titer ≥ 40) are reported in the text and discussion for clarity.

### 2.3. Statistical Analysis

Genre and species variables were screened using a chi-square test to check for statistically significant associations with serological status. Data were analyzed using chi-square statistics in R [13].

## 3. Results

### 3.1. Bat Collection and Trapping Localities

A total of 329 bats were collected in 12 different locations (Figure 1). The majority (*n* = 154) belonged to the Vespertilionae family in which six different species were represented. All bats of the Miniopteridae (*n* = 132) and Molossidae (*n* = 35) family belonged to the Schreiber’s long-fingered bat (*Miniopterus schreibersii*) and European free-tailed bat (*Tadarida teniotis*) species, respectively.

### 3.2. Serological Analysis

Among the 315 sera tested for the presence of NT-Ab against SFSV, 11 (3.5%) were positive (cut-off ≥ 40), in 3 of the 12 localities where the bats were sampled (refuges 4, 7, and 11, Figure 1, Table 1 and Table 2, and Table S1 of supplementary data). Among the 170 sera tested for the presence of NT-Ab against TOSV, 8 (4.7%) were positive (cut-off ≥ 40), in 4 of the 12 localities where the bats were sampled (refuges 4, 7, 11, and 12, Figure 1, Table 1 and Table 2). None of the bats were found to be positive for both viruses (cut-off ≥ 40). All bats captured in hibernation refuges (refuges 1 and 2, Figure 1, Table 1 and Table 2) were seronegative for both viruses. All seropositive samples were obtained in summer or equinoctial colonies. Positive sera were detected for both viruses in common serotine (*Eptesicus serotinus*), Schreiber’s long-fingered bat (*M. schreibersii*), and mouse-eared Myotis (*Myotis myotis*)*;* European free-tailed bat (*T. teniotis*) sera were positive for TOSV only (cut-off ≥ 40) (Table 1). Common serotine (*E. serotinus*) had the higher seroprevalence for SFSV (22.6%) and TOSV (10%) (Table 1 and Table 2). Seroprevalence differences for SFSV were found among common serotine (*Eptesicus serotinus*), Schreiber’s long-fingered bat (*M. schreibersii*), and mouse-eared Myotis (*Myotis myotis*)*;* European free-tailed bat (*T. teniotis*) (SFSV χ^2^ = 28.94, df = 2, *p* < 0.001). We have not found differences in SFSV and TOSV prevalence between females and males (SFSV χ^2^ = 2.07, *p* > 0.1; TOSV χ^2^ = 0.01, *p* > 0.1) (Table 3). Among the eight different species tested, four did not show any positive samples for SFSV nor TOSV (Savi’s pipistrelle (*Hypsugo savii*)*,* long-fingered Myotis (*Myotis capaccinii*)*,* Escalera’s bat (*Myotis escalerai*)*,* and common pipistrelle (*Pipistrellus pipistrellus*)).

## 4. Discussion

Pathogens transmitted to humans and animals by phlebotomine sandflies are relatively neglected, although related diseases are prevalent and emerging in large areas of southern Europe [4]. The actual incidence of phlebovirus infections in the Mediterranean area is unknown, although at least 250 million people are potentially exposed [1]. The recent geographical expansion of sandflies competent for phleboviruses in the Mediterranean region has been attributed to climate changes, since temperature is a major determinant for the activity of phlebotomine flies [14]. Accordingly, this justifies carrying out eco-epidemiological studies to better understand the dynamics of medically important viruses such as TOSV and SFSV, and the role potentially played by vertebrates present in the areas where populations are exposed and specifically by species such as bats. The reasons for suspecting that bats play a role in the ecology of sandfly-borne phleboviruses are multiple: (i) TOSV was isolated once from the brain of a dead Kuhl’s pipistrelle (*Pipistrellus kuhlii*) in Italy in an area where multiple strains of TOSV were found in sandflies [15]; (ii) bats are living in an environment where they are in close contact with phlebotomine flies (caves, mines, attics, cracks); (iii) they can get infected after the bite of an infected female sandfly, and they also can get infected by eating sandflies as other arboviruses which have been shown to cause infection through an oral route (tick-borne encephalitis virus, Alkhurma hemorrhagic fever virus [16,17,18]); and (iv) bats are a reservoir for a large number of zoonotic viruses (coronaviruses, flaviviruses, astroviruses, paramyxoviruses, and filoviruses [7,19]). Thus, it is important to investigate if bats can be a reservoir or play a role in the amplification of the circulation of these viruses.

To the best of our knowledge, this study is the first to investigate sandfly-borne phleboviruses in European bat colonies. Trapping locations were selected in this geographic area because of the previous reports of the endemic presence of multiple sandfly-borne phleboviruses such as TOSV, Arrabida virus, Massilia virus, or Granada virus [20,21,22,23,24]. There were positive sera in all three families included in the study (Vespertilionidae, Miniopteridae, and Molossidae). Bat species showing exposure to TOSV or SFSV present a large distribution in Mediterranean basin (IUCN red list 2019: https://www.iucnredlist.org/assessment/red-list-index). Of the six species belonging to the Vespertilionidae family, only two species (common serotine (*Eptesicus serotinus*) and mouse-eared Myotis (*Myotis myotis*)) were seropositive for both viruses. The other four species of this family (Savi’s pipistrelle (*H. savii*)*,* long-fingered Myotis (*M. capaccinii*)*,* Escalera’s bat (*M. escalerai*)*,* and common pipistrelle (*P. pipistrellus*)) were negative for both viruses although the absence of positive results could be due to the low number of samples tested. Common serotine (*E. serotinus*) is a synanthropic species that live in attics, roofs, and cracks of houses, while mouse-eared Myotis (*M. myotis*) from the Mediterranean region usually live in caves. These two species show gregarious behavior during the breeding period. The mouse-eared Myotis (*M. myotis*) from Majorca form mixed-breeding colonies with Schreiber’s long-fingered bat (*M. scheirbersii*); this species belongs to the Miniopteridae family and was also seropositive. Schreiber’s long-fingered bat (*M. schreibersii*) is a migratory bat with strong gregarious behavior that lives in caves or mines. Some works suggest that the migratory species of bats may contribute to the spatial diffusion and maintenance of viruses [25,26]. However, the role of Schreiber’s long-fingered bat (*M. schreibersii*) in the dynamics of sandfly-borne phleboviruses is unknown. The European free-tailed bat (*T. teniotis*) belongs the Molossidae family and is the fourth species that we found to be seropositive. This gregarious species take refuge in cracks of the cliff. Although two were positive for TOSV at titers equal to or greater than 40, four individuals had titers at equal to or greater than 20 for SFSV.

Unlike the other two species, most of the positive sera of Schreiber’s long-fingered bat (*M. schreibersii*) and the European free-tailed bat (*T. teniotis*) had low titers (≤40). Determining whether these differences are a consequence of ecological, immunological, or phylogenetic factors is very difficult. Perhaps the phylogenetic distance between the Vespertilionidae, Miniopteridae, and Molossidae contributes to these differences [27], as was shown in studies on coronaviruses and lyssavirus in bats [28,29]. However, further studies are needed to investigate this hypothesis.

Whether the cut-off value for positivity is 20 or 40 is difficult to solve; however, the average percentage of positive samples is not different with either of the criteria for positivity (3.5% vs. 5.7% for SFSV and 4.7% vs. 5.3% for TOSV). Since the average life expectancy of bats is 10–15 years, these figures do not support the theory that bat colonies act as a reservoir for TOSV or SFSV; indeed, if this were the case then a much higher prevalence would have been expected. Aside from the results of the current study, we have been testing bat tissue samples (mainly blood) for the presence of TOSV and SFSV RNA: they have been continuously negative, and although these negative results have not been published to date we believe that they are of interest to be mentioned here (Serra-Cobo and Charrel, unpublished results).

The refuges can have different ecological functions: hibernation, equinoctial, and breeding. The hibernation refuges shelter bats in winter and have low temperatures ranging from between 6–9 °C: the body temperature of hibernation bats is only 1 or 2 °C above the temperature of the refuge. The breeding refuges shelter bats at the end of spring and during summer, usually have high temperatures (more than 20 °C), and are located near areas with abundant insects. The equinoctial refuges shelter bats at the end of winter and during spring and autumn and have intermediate temperatures (14–18 °C). There were no seropositive bats in the two hibernation colonies of Schreiber’s long-fingered bat (*M. schreibersii*). Several bat viruses, including lyssaviruses, exhibit a strong seasonal pattern of viral dynamics during the breeding period favoring bat infection [7,30,31]. Temperature is also a major determinant for the seasonal interval of sandfly activity; they are not active at low temperatures [14]. This information is in concordance with our results. There were also three localities where bats were dually positives for TOSV and SFSV (refuges 4, 7, and 11), whereas only TOSV-positive sera were found in refuge 12. The dual positivity observed in three species and in three localities suggests that there is not a very high specificity in terms of environment for a given species of virus, and that possibly the same vector may be able to transmit two different viruses (this point has already been raised in previous studies which show a more relaxed virus–vector relationship than previously believed) [32]. The absence of sera reacting with both TOSV and SFSV confirm the lack of cross-reactivity, and the fact that NT-Abs are specific to each virus, namely TOSV and SFSV.

Although is still too early to determine the role of bats in the dynamics of phleboviruses considering that there is no evidence that bats are playing a role in the natural cycle of either TOSV or SFSV, studying large breeding colonies of bats can be used as a sentinel for surveillance of the presence of such viruses in a given locality. This is important when considering the geographical expansion of sandflies competent for phlebovirus in the Mediterranean region.

## Figures and Tables

**Table 1:** Distribution of tested specimens per bat species and presence of neutralizing antibodies against SFSV and TOSV.

	Tested for SFSV			Tested for TOSV		
Species	Total	Pos ^a^	% ^b^	Total	Pos ^a^	% ^b^
*Vespertilionidae*	149	10 (10)	6.7	103	5 (5)	4.8
*Eptesicus serotinus*	31	7 (7)	22.6	30	3 (3)	10.0
*Hypsugo savii*	8	0	0.0	0	nt	nt
*Myotis capaccinii*	16	0	0.0	16	0	0.0
*Myotis escalerai*	3	0	0.0	3	0	0.0
*Myotis myotis*	90	3 (3)	3.3	54	2 (2)	3.7
*Pipistrellus pipistrellus*	1	0	0.0	0	nt	nt
*Miniopteridae*						
*Miniopterus schreibersii*	131	1 (4)	0.8 (3.0)	34	1 (2)	2.9 (5.9)
*Molossidae*						
*Tadarida teniotis*	35	0 (4)	0.0 (11.4)	34	2 (2)	5.9
TOTAL	315	11 (18)	3.5 (5.7)	170	8 (9)	4.7 (5.3)

^a^, number of positive sera when cut-off titer ≥ 40 (when cut-off titer ≥ 20); ^b^, percentages are calculated when number of sera is ≥ 100; nt, not tested.

**Table 2:** Distribution of tested specimens per locality and presence of neutralizing antibodies against SFSV and TOSV.

				SFSV			TOSV		
No	Locality	Status	Species (nb)	Total	Pos^a^	%^b^	Total	Pos^a^	%^b^
1	Ferreries	H	*M. schreibersii* (30)	30	0	0.0	1	0	0.0
2	St. Llorenç Savall	H	*M. schreibersii* (33)	33	0	0.0	2	0	0.0
3	Binifaldó	F	*H. savii* (8), *P. pipistrellus* (1), *T. teniotis* (1)	10	0	0.0	nt	nt	nt
4	Olesa de Bonesvalls	E-B	*M. schreibersii* (24)	24	1 (1)	4.2	15	1 (1)	6.7
5	Ciutadella	E	*M. schreibersii* (3)	3	0	0.0	nt	nt	nt
6	Palma de Mallorca	E	*M. myotis* (2)*, M. schreibersii* (3)	5	0	0.0	2	0 (1)	0.0 (50.0)
7	Navarcles	B	*E serotinus* (31)	31	7 (7)	22.6	30	3 (3)	10.0
8	Malgrat de Mar	E-B	*M. schreibersii* (9)	9	0 (1)	0.0 (11.1)	1	0	0.0
9	Calvià	B	*M. myotis* (27)*, M. schreibersii* (7)	34	0 (1)	0.0 (2.9)	5	0	0.0
10	Inca	B	*M. myotis* (40)*, M. capaccinii* (16), *M. escalerai (3)*, *M. schreibersii* (10)	63	0	0.0	69	0	0.0
11	Llucmajor	B	*M. myotis* (67)*, M. schreibersii* (13)	39	3 (4)	7.7 (10.3)	11	2 (2)	18.2
12	Oliete	B	*T. teniotis* (34)	34	0 (4)	0.0 (11.8)	34	2 (2)	5.9
	TOTAL			315	11 (18)	3.5 (5.7)	170	8 (9)	4.7 (5.3)

H, hibernation; F, forestry; E, equinoctial; B, breeding; ^a^, number of positive sera when cut-off titer ≥ 40 (when cut-off titer ≥ 20); ^b^, percentages are calculated when number of sera is ≥100; nt, not tested.

**Table 3:** Serological results by sex.

	SFSV			TOSV		
Sex	Total	Positives ^a^	%	Total	Positives ^a^	%
Females	203	9 (15)	4.4 (7.4)	126	7 (7)	5.5 (5.5)
Males	112	2 (3)	1.8 (2.7)	44	1 (2)	2.2 (4.4)
TOTAL	315	11 (18)	3.5 (5.7)	170	8 (9)	4.7 (5.3)

^a^, number of positive sera when cut-off titer ≥ 40 (when cut-off titer ≥ 20).

## Data Availability

Data are contained within the article or supplementary material.

## Supplementary Materials

The following are available online at https://www.mdpi.com/1999-4915/13/1/88/s1, Table S1: Positive sera for SFSV NT Abs.
