# Supplementary material for: Seroprevalence of Toscana Virus and Sandfly Fever Sicilian Virus in European Bat Colonies Measured Using a Neutralization Test
Source: Viruses. 2021 Jan 11;13(1):88. doi: 10.3390/v13010088 (PMC7826795; doi:10.3390/v13010088)
Supplement: Supplementary file 1 [file viruses-13-00088-s001.pdf]

Table S1. Supplementary data. Positive sera for SFSV NT Abs

Number	Titre	Species	Sex	Locality	Date
50	1/320	*Eptesicus serotinus*	f	Navarcles	6/6/07
59	1/320	*Eptesicus serotinus*	f	Navarcles	6/6/07
60	1/80	*Eptesicus serotinus*	f	Navarcles	6/6/07
65	1/80	*Eptesicus serotinus*	f	Navarcles	6/6/07
67	1/320	*Eptesicus serotinus*	f	Navarcles	6/6/07
77	1/160	*Eptesicus serotinus*	f	Navarcles	6/6/07
79	1/320	*Eptesicus serotinus*	f	Navarcles	6/6/07
27	1/20	*Miniopterus schreibersii*	f	Palomeres	23/12/11
67	1/20	*Miniopterus schreibersii*	f	Esquerda	5/7/17
116	1/20	*Miniopterus schreibersii*	f	Sa Guitarreta	8/7/17
108	1/40	*Miniopterus schreibersii*	f	Esquerrà	19/5/18
97	1/160	*Myotis myotis*	m	Sa Guitarreta	3/8/07
99	1/40	*Myotis myotis*	f	Sa Guitarreta	3/8/07
100	1/160	*Myotis myotis*	m	Sa Guitarreta	3/8/07
26	1/20	*Tadarida teniotis*	f	San Pedro	10/6/06
34	1/20	*Tadarida teniotis*	m	San Pedro	10/6/06
42	1/20	*Tadarida teniotis*	f	San Pedro	10/6/06
44	1/20	*Tadarida teniotis*	f	San Pedro	10/6/06
